# Intracranial infection caused by *Mycobacterium rhodesiae* with specific imaging findings and good response to medication: a case report and literature review

**DOI:** 10.3389/fmed.2024.1414369

**Published:** 2024-05-30

**Authors:** Siwei Chen, Wei Sun, Ran Liu, Lanqiu Yao, Qing Peng

**Affiliations:** ^1^Department of Neurology, Peking University First Hospital, Beijing, China; ^2^Magma Capital Funds, Chicago, IL, United States

**Keywords:** nontuberculous mycobacteria, central nervous system infection, encephalitis, next-generation sequencing, extrapulmonary manifestation

## Abstract

Nontuberculous mycobacteria (NTM) are exceedingly rare etiological agents of intracranial infections. Among them, *Mycobacterium rhodesiae* stands out as an even less common pathogen. In this paper, we report the first documented case of a central nervous system (CNS) infection in humans caused by *Mycobacterium rhodesiae*, which has specific imaging findings and good response to the therapy by using Linezolid, Clarithromycin, and Minocycline. The diagnosis was facilitated by a comprehensive multimodal approach, incorporating multisite imaging, cerebrospinal fluid analysis via next-generation sequencing (NGS), and targeted genetic testing. Furthermore, this paper provides a derivation of the clinical characteristics observed in other documented instances of CNS infections attributable to NTM and based on a review of the current literature. Our experience contributes to the evidence that is needed to understand the full spectrum of NTM-related CNS pathologies and underscores the importance of a multidisciplinary diagnostic process in atypical presentations of intracranial infections.

## Introduction

Central nervous system (CNS) infections caused by nontuberculous mycobacteria (NTM) present significant diagnostic challenges, often leading to misdiagnosis and inappropriate treatment with conventional antitubercular drugs (1). Intracranial NTM infections, in particular, can manifest with sudden onset of focal neurological deficits, without typical symptoms like fever or headache, mimicking stroke, and necessitating differentiation from other diseases. The existing literature primarily focuses on pulmonary manifestations of NTM, leaving a gap in established guidelines for CNS-related NTM infections.

NTM species known to cause CNS infections can be classified into four groups based on their growth rate, morphology, and pigmentation response to light (2). Among these, *Mycobacterium rhodesiae*, a rapid-growing scotochromogenic mycobacterium, was first identified in 1971 from the sputum of Rhodesian patients suspected of pulmonary tuberculosis (3). Infections outside the lungs, especially caused by *Mycobacterium rhodesiae*, are extremely rare. To date, only one case of peritonitis has been attributed to this pathogen (4). In this paper, we report the first case of intracranial infection caused by *Mycobacterium rhodesiae*, enriching the medical literature by illustrating a novel manifestation of NTM infections.

## Case presentation

**Figure 1:**
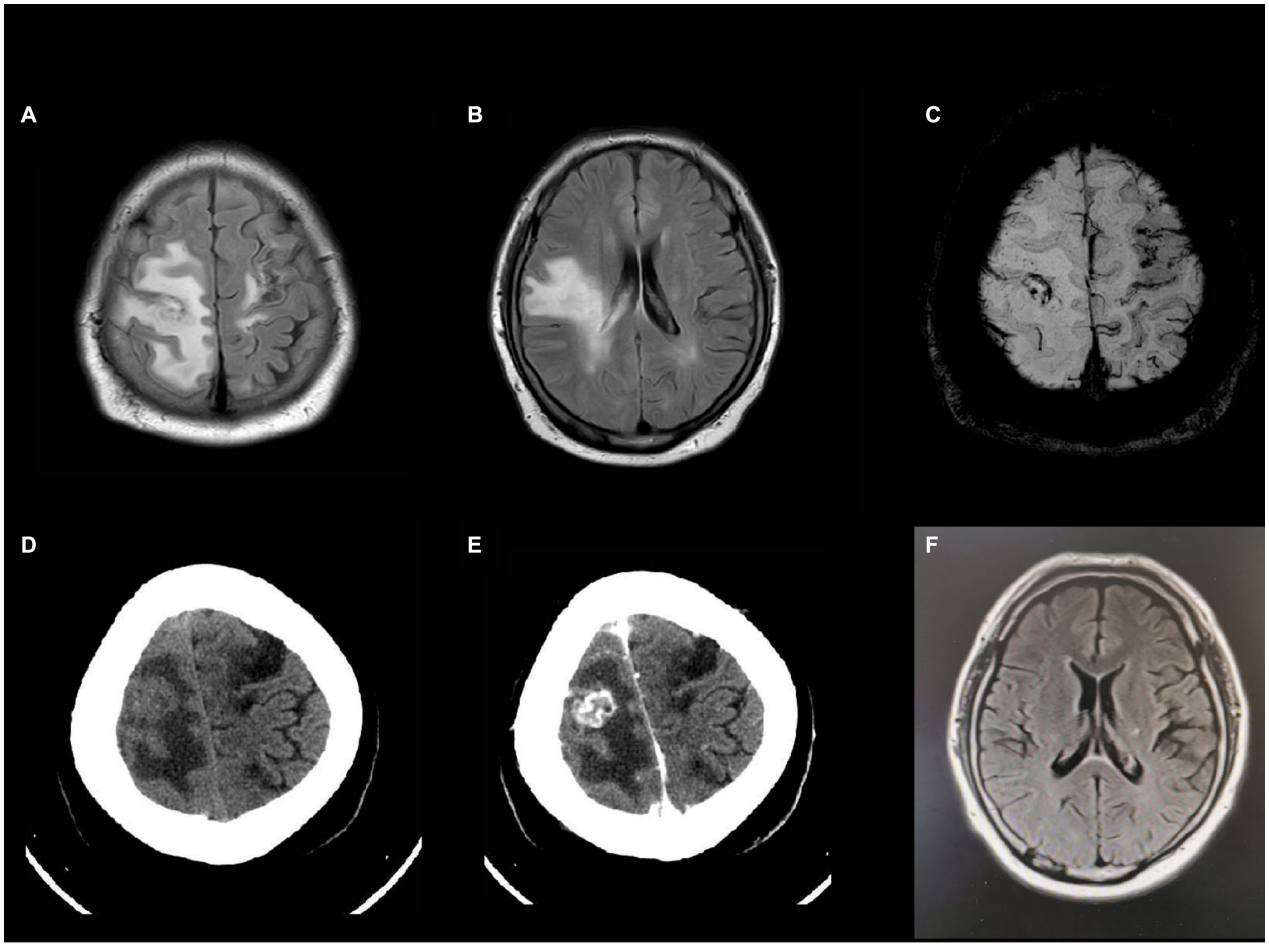
Head MRI and enhanced CT after admission **(A,B)** T2WI showed expansion of white matter hyperintensity with mixed signals inside. **(C)** Susceptibility weighted imaging (SWI) indicated microbleeds at both new lesions and old infarctions. **(D,E)** Hypodensity lesions in head CT scan and rim-like or nodule-like enhancement inside the lesion in enhanced CT. **(F)** Head MRI 18 months later showed that the extent of lesion had regressed significantly.

**Figure 2:**
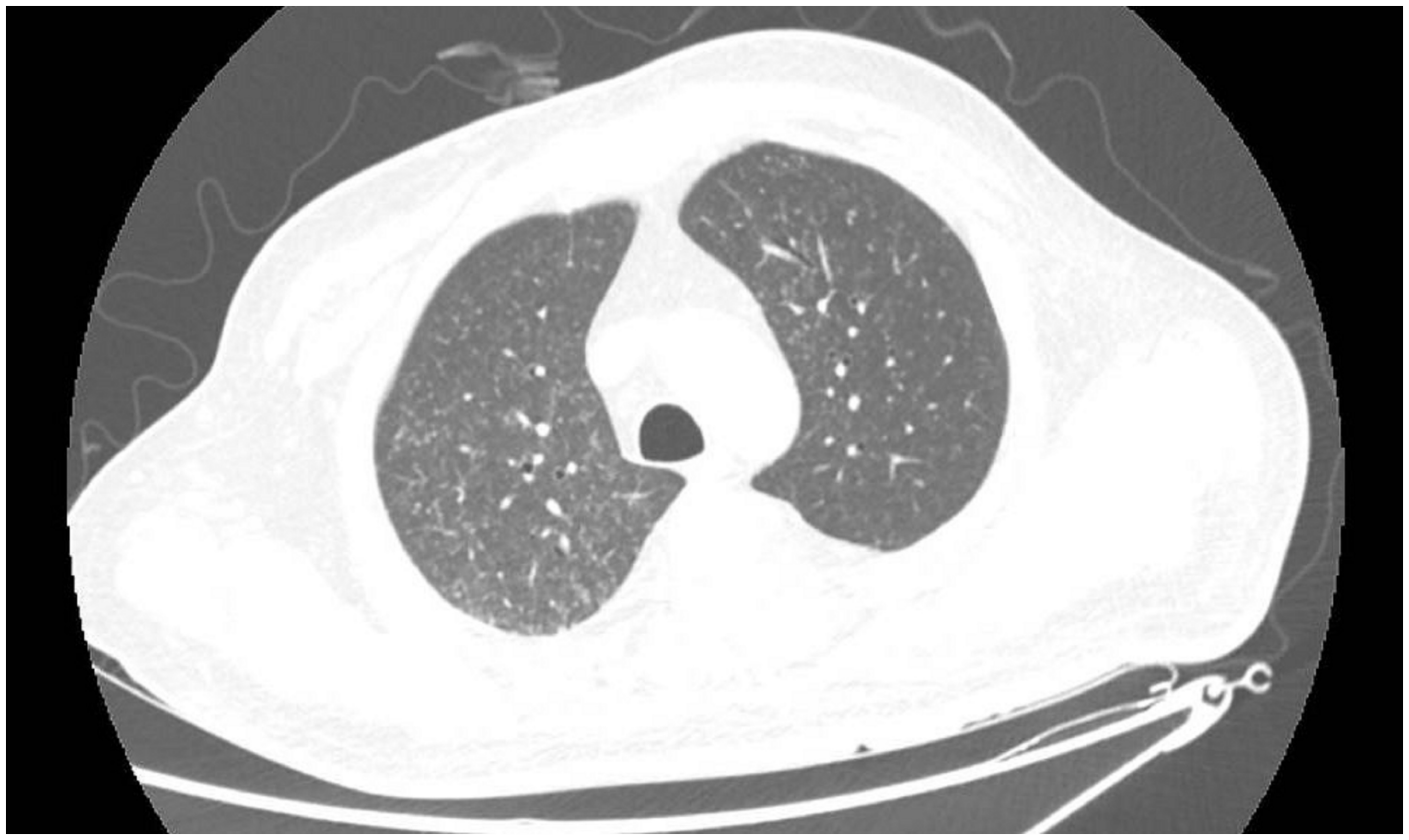
Chest CT scan on admission indicated diffused miliary nodules in bilateral lungs.

A 41-year-old male working as a sanitation worker had two episodes of stroke 10 months before admission in 2021, exhibiting sudden onset of numbness and weakness in his right limbs. He was treated with aspirin and clopidogrel. The patient’s modified Rankin Scale (mRS) reached grade 2 after discharge. The patient was diagnosed with Type-2 diabetes and acute tubulointerstitial nephritis during his first hospitalization. Prednisone was started to treat nephritis. Three months before admission, he had a serious lung infection with a maximum temperature of 39 degrees Celsius and abnormal chest imaging. Fiberoptic bronchoscopy and next-generation sequencing (NGS) examination of the alveolar lavage solution suggested nontuberculous mycobacterial (NTM) infection. Azithromycin and rifampicin were given for the treatment of NTM but were stopped because of gastrointestinal adverse effects. Subsequently, the patient was administered clarithromycin and linezolid to treat NTM till his temperature returned to normal range. One month before admission, he experienced a sudden onset of weakness and numbness in his left limbs, however, he had no headache or fever. The magnetic resonance imaging (MRI) of the head showed newly occurred abnormal signals in the right frontal lobe with possible bleeding and without any obvious abnormality in cranial arteries or veins. The peritoneal dialysis was initiated for his increasing creatinine levels. The patient’s weakness worsened gradually, the muscle strength of his left limbs decreased to level 3, and his mRS was grade 4. In the interim, he began to experience episodic tics in his left limbs. After admission, the MRI (Figures 1A,B) showed mixed signal lesions in the right frontal and parietal lobe with significant edema around the lesions. The microbleeds could be seen in both new lesions and old infarctions in the brain (Figure 1C). The computed tomography (CT) of head showed iso-density lesions in white matter and low-density lesions around the right frontal and parietal lobe with rim-like or nodule-like enhancement in the frontal parietal lobe (Figures 1D,E). The Magnetic resonance spectroscopy pattern of the lesions showed decreased N-Acetylaspartic acid (NAA) peak, an increased choline peak, and the presence of lactate peak and lipid peak. The cerebrospinal fluid (CSF) revealed normal cranial pressure and cell count (2 × 10^6^ cells/L) with elevated protein level (0.97 g/L) and decreased glucose level (3.52 mmol/L). His blood glucose level was 12.4 mmol/L. The Acid-fast staining test for bacteria was negative. The Adenosine deaminase level in CSF was 1.6 U/L. However, the NGS of the CSF detected *Mycobacterium rhodesiae* which provided evidence for infection of the central nervous system. The cytology of CSF did not reveal tumor cells. Furthermore, the chest CT scan showed diffuse small nodules in bilateral lungs (Figure 2) that were similar to his previous chest CT scan 3 months earlier, indicating a high possibility of lung infection.

From a clinical perspective, for a 41-year-old young man who had several episodes of stroke history with unusual imaging features, other etiological factors should be differentiated. First, primary angiitis of the central nervous system (PACNS) should be differentiated as the patient had multiple infarctions with microbleeds at both new lesions and old infarctions. The PACNS can also have tumor-like appearance with predominant edema around. Second, the hereditary cerebral small vessel diseases (hCSVD) should be excluded. Third, the tumors should be considered.

Further examinations were conducted which included tests for blood tumor markers, autoimmune antibodies, and a genetic test for hCSVD, with all negative results for these examinations. The color fundus photography indicated scattered hemorrhages and exudates in both eyes.

Linezolid, clarithromycin, and minocycline were administered for 6 months. The patient’s symptoms improved and brain lesions diminished gradually. However, the patient stopped taking the medications after 3 months, due to gastrointestinal adverse effects. After a two-year follow-up, the patient did not have any more stroke-like episodes or seizures. After 18 months, the MRI of head showed the extent of the lesion had regressed significantly (Figure 1F). After rehabilitation, the patient’s mRS improved to grade 2.

## Discussion and literature review

NTM are widely present in soil and water. They are known to cause insidious skin and soft tissue infections after trauma or surgery, chronic lung diseases, and disseminated disease in the immunosuppressed hosts (5). Intracranial infection caused by NTM is a very uncommon occurence. The cases reported in the literature are mainly related to the retention of intracranial hardware in neurosurgery or individuals with immunosuppressed conditions such as human immunodeficiency virus (HIV) infection (6–14). Furthermore, there are some cases related to trauma, especially traumatic head injury including facial fracture and facial gunshot injury (5, 15, 16). All these patients with a history of trauma had undergone surgery related to their cranial injuries.

There are different NTM species mentioned in the literature that can cause intracranial infection, among which, *Mycobacterium avium complex* is the most common species for cause of NTM-CNS infection, followed by *Mycobacterium fortuitum* and *Mycobacterium abscessus* (17). The other rare species that can infect CNS include: *Mycobacterium kansasii*, *Mycobacterium haemophilum*, *Mycobacterium mucogenicum*, *Mycobacterium neoaurum*, *Mycobacterium houstonense*, etc.

*Mycobacterium rhodesiae* is an exceptionally uncommon species of NTM and only one relevant literature of extrapulmonary infection has reported its ability to induce peritonitis in patients with continuous ambulatory peritoneal dialysis (4). The peritonitis of the patient was cured by administering ciprofloxacin and clarithromycin for 3 months. Based on our knowledge, the present case is the first reported case of intracranial infection caused by *Mycobacterium rhodesia* in literature. In this case, the patient who had a prior lung infection, the risk of developing an intracranial infection was higher as compared to the individuals without such a history. The probable source of infection was speculated to be the hematogenous dissemination from the pulmonary infectious foci. Furthermore, the breakdown of the blood–brain barrier following cerebral infarction had increased the risk of intracranial infection. Although the patient had continuous peritoneal dialysis, he did not exhibit abnormal abdominal symptoms, despite the risk of the presence of mycobacterium in dialysate fluid. Furthermore, the multiple peritoneal dialysis tests were within the normal range. The diabetes and the immunosuppression due to prednisone use, could have been the triggering factors for the opportunistic infection in the patient. Furthermore, working as a sanitation worker also increased the risk of infection.

Identifying the nature of the patient’s intracranial lesions was a major challenge during his hospitalization. Our patient was a 41-year-old man exhibiting signs of focal neurologic defects. The patient did not have a fever, headache, or any manifestations of increased intracranial pressure on admission. In addition to the patients’ history of stroke in recent months and unusual imaging features, PACNS and hCSVD as well as the tumor should be differentiated. The brain imaging showed patchy white matter lesions as well as rim-enhancing lesions, which could mimic brain imaging of PACNS (18) and the retinal vasculopathy with cerebral leukoencephalopathy and systemic manifestations (RVCL-S, a special type of hCSVD) (19, 20). Hedderich et al. revealed that the periventricular patchy white matter lesions in the frontal lobes and the rim-enhancing lesions with prolonged diffusion restriction and long-lasting contrast enhancement were characteristic imaging findings in RVCL-S and could be helpful in the differential diagnosis (19). Furthermore, the patient also had retinopathy and renal injury. Therefore, RVCL-S needed to be differentiated. However, the patient had no relevant family history. The renal injury in patient was due to tubulointerstitial nephritis rather than vascular nephropathy, and his genetic test did not support hCSVD. As for PACNS, it could not be clearly diagnosed without biopsy or exclusion of other possible diseases. Intracranial infection may also cause some infectious vasculitis that resembles PACNS. It was speculated that the patient’s cerebral infarction could be due to diabetic small artery occlusion. The presence of retinopathy, on fundoscopic examination, and the tubulointerstitial nephritis both possibly due to diabetes further supported this diagnosis. Besides, the brain tumor should also be differentiated for the patient had abnormal contrast enhancement for these foci and his MRS showed increased Cho peak, with presence of lactate and lipid peaks. However, his CSF results and therapeutic response excluded this diagnosis.

To date, diagnosing CNS NTM infection still poses a big challenge for clinical practice because of its rare occurrence due to its rarity and there may be some possibility of atypical manifestations. The rarity of *Mycobacterium rhodesiae* as an etiological agent might also be due to the limitation of detection technology and limited clinical awareness. However, with the improvement of detection technology, NGS has greatly helped the diagnosis of *Mycobacterium rhodesiae* infection. Therefore, we reviewed the literature that reported intracranial NTM infection with detailed clinical data published from 1993 to 2023 and summarized the features of the diseases including the susceptible population, clinical characteristics, imaging findings, treatment effects, etc.

In our review of the literature, cases span from the 1960s to the present. By the end of December 2023, there were 114 cases of intracranial NTM infection reported or summarized in the literature (5, 6, 8–16, 21–27). Antonia Flor et al. reported two cases and identified 50 cases of NTM meningitis in the English-language literature before 1994 (8). Among these cases, limited clinical information was available. The case reports over the past 20 years provided us with more detailed clinical data.

NTM species that are known to cause CNS infections can be categorized into two primary groups based on their growth rate: slow-growing mycobacteria (SGM) and rapid-growing mycobacteria (RGM). For a more detailed classification, these species can be further subdivided into four categories. These include photochromogens (SGM), scotochromogens (SGM), nonphotochromogens (SGM), and RGM, distinguished by their growth rate, morphology, and pigmentation changes in the presence of light. This classification system was initially established by Runyon, offering a comprehensive framework for understanding NTM species in the context of CNS infections (2). Out of the 114 cases, 39 were caused by *Mycobacterium avium complex*, 24 by *Mycobacterium fortuitum group*, 18 by *Mycobacterium abscessus*, 7 by *Mycobacterium haemophilum*, 6 by *Mycobacterium massiliense*, 4 by *Mycobacterium kansasii*, 4 by *Mycobacterium mucogenicum*, 4 by *Mycobacterium chelonae*, and 3 by *Mycobacterium goodii*. The rare cases included one infection each of *Mycobacterium neoaurum*, *Mycobacterium houstonense*, *Mycobacterium bolletii*, *Mycobacterium immunogenum*, and *Mycobacterium rhodesiae* (our case).

The age distribution of patients was from 9 months to 97 years, and the disease can occur in all ages. The ratio of male to female patients was approximately 1.6:1. Approximately 30% of these cases had the underlying condition of HIV infection. Eight patients were on long-term glucocorticoid therapy, which might have intensified their immunosuppressed condition. The other common procedure that may induce intracranial NTM infection is neurosurgical hardware implantation, especially ventriculoperitoneal shunt, and lumbar peritoneal shunt (6, 13–15, 21, 24, 28). The other surgeries include ocular prosthesis implantation (5), aortic valve replacement (29), and cranioplasty (16). It was uncertain whether performing lumbar puncture in immunosuppressed patients could lead to intracranial NTM infection (12). Among the 114 cases, the most common species related to neurosurgical hardware implantation was the *Mycobacterium fortuitum group*, and patients with intracranial infection caused by *Mycobacterium haemophilum* were all combined with AIDS.

The clinical manifestations of these patients varied. In the normal conditions, the patients would exhibit infectious meningitis or meningoencephalitis, including fever, headache, dizziness, nausea, vomiting, and neck stiffness. A few patients exhibited behavioral disorder, decrease in vision, hemiplegia, epilepsy, and change in mental status. However, a small number of patients may have atypical clinical symptoms like progressive cognitive impairment or subcutaneous abscess (15, 22, 24, 30), whereas some patients can have no signs of infection and even be asymptomatic (16).

CSF findings were not reported in all case reports. However, from among those cases that provided CSF results, the values varied significantly. Patients may have normal intracranial pressure. They usually had a mild to moderate elevated white cell count, low glucose level, and a high protein level. However, not every patient can have such a typical cerebrospinal fluid that may indicate CNS infection. In some patients, the cerebrospinal fluid may be completely normal. From cases and literature review, white cell count ranged from 0 to 3,025 × 10^6^ (cells/L), lymphocytes in predominant. However, *Mycobacterium abscessus* and *Mycobacterium fortuitum* infection can cause CSF polymorphonuclear leucocytes increased predominantly. The glucose level in CSF can be normal or decreased compared to serum glucose. The protein level ranged from 0.33 to 3.9 g/L. The positive rate for acid-fast bacterial smear in CSF did not exceed 30%. However, for those patients who had negative results of acid-fast bacterial smear, acid-fast bacterial culture may be positive.

With the improvement of detection methods, the application of NGS increases the detection rate of intracranial NTM infection and helps clinical diagnosis. Although NTM detection in some samples may result from contamination, identifying NTM in sterile fluids like CSF is critical (5). This finding should be taken into consideration, particularly when a patient is suspected of having NTM infection at other sites, or when there is no evidence of the presence of other pathogens.

The primary symptoms of patients affected by NTM infection in CNS are predominantly meningitis and meningoencephalitis, with ventriculitis also being reported. The rarely reported lesions include arteritis (30), cerebral thrombophlebitis (31), optic tract (9), and spinal cord (25). To date, no fixed or summarized description of brain imaging for NTM infection of the central nervous system is available. From among the cases that we reviewed, some articles provided detailed description of brain or spinal cord imaging (5, 6, 9–11, 16, 24–26). Most of the lesions showed T2 hyperintensity with enhancement in T1 postcontrast imaging. They can be multifocal patchy white matter lesions, or nodular masses, or rim-like lesions, which may be similar to the images of tuberculosis infection (17, 32). Some lesions can mimic cerebrovascular disease or tumors with mild edema (26). The CT scan may present as iso-density or low-density lesions in white matter, and with contrast, there can be ring enhancement similar to the MRI findings. For ventriculitis, both the MRI and CT scans can have significant ring-enhancing or high-density lesions along the ventricles. For our case, we also consulted infectiologists and radiologists for accurate diagnosis. Therefore, we speculated that multifocal nodular or rim-like enhancement lesions with patchy white matter and T2 hyperintensity could be certain specific imaging presentations of NTM infectious encephalitis.

Patients with intracranial NTM infection may also have concomitant NTM foci in other systems, which might contribute to the final diagnosis of CNS infection. The extra-CNS lesions mainly occur in the soft tissue of unhealing wounds or around the surgical hardware, and the abdomen (6, 13, 15, 21, 24, 28), the other places include the pulmonary system, endocardium liver, and bone marrow (15, 16, 29, 31, 33).

Treating CNS infections caused by NTM is extremely challenging, given the scarcity of literature and lack of robust research evidence. The treatment of NTM is mostly based on observational studies, as there are no comprehensive clinical trials in this area (1). Furthermore, there are no studies on the efficacy of different antibiotics for different subspecies of NTM. However, one of the important principles in managing CNS infection caused by NTM is identification of the source of infection, adequate drainage of abscesses, and removal of foreign material (5). In serious infections, initial treatment typically involves two or three drugs followed by a prolonged course of oral medication, especially for immunosuppressed patients or those with a persistent infection focus (5). Clarithromycin, azithromycin, rifabutin, ethambutol, amikacin, or streptomycin and levofloxacin or moxifloxacin can be used (34). In addition to these drugs, clarithromycin and azithromycin, macrolides with moderate blood–brain barrier penetration have better suitability for patients with renal impairment due to liver metabolism. Rifabutin offers effective blood–brain barrier penetration but necessitates caution in renal dysfunction. Ethambutol, with limited penetration, requires careful use in renal impairment. Aminoglycosides, such as amikacin and streptomycin, present higher risks for renal-impaired patients due to low penetration and renal excretion. Levofloxacin and moxifloxacin, the fluoroquinolones, effectively cross the blood–brain barrier, but levofloxacin requires renal adjustment as it is not completely excreted by the kidneys. Minocycline has a relatively high penetration into the central nervous system. This characteristic has made it an area of interest in the treatment of various neurological conditions and diseases, as it allows the drug to exert its effects within the brain tissue. A newer agent, linezolid, was reported to have excellent activity against NTM with excellent penetration into the CNS and can be given orally (35, 36). Our patient, who had renal failure, was treated with a combination of linezolid, clarithromycin, and minocycline for 3 months, resulting in a favorable outcome.

Intracranial NTM infection has a high mortality rate. The total mortality rate is up to 38%. However, the outcomes can vary depending on the species, suggesting differences in the pathogenic power of these organisms. In general, patients with *Mycobacterium fortuitum* infection tend to have a lower death rate and may exhibit less drug resistance when compared to those with *Mycobacterium abscessus* (5). Previous literature also indicated that a high CSF protein level is a risk factor for poor outcomes (27). However, further exploration and data accumulation is required to know whether intracranial infection caused by *Mycobacterium rhodesiae* could have a better outcome and exhibit less drug resistance as compared to other species. For this patient who had renal failure, choices of treatment were limited and challenging. However, after a 2-year follow-up, the patient’s condition stabilized, which suggests that the combination of linezolid, clarithromycin, and minocycline could be a viable treatment strategy for intracranial NTM infection in patients with renal failure. However, it is important to note that the conclusions drawn from a single case should be approached with caution, and further research is needed to confirm the best therapeutic strategies and to evaluate the efficacy of existing anti-mycobacterial drugs against *Mycobacterium rhodesiae*. Further research could focus on molecular techniques that can rapidly and accurately detect this bacterium, saving more time for accurate diagnosis and prompt treatment.

## Conclusion

There are many species of NTMs and the nomenclature of different species is constantly updated with advances in microbiology research. It is important to recognize that once NTM is detected, especially in sterile fluids such as cerebrospinal fluid, it needs to be considered with caution. The intracranial NTM infection can present as a sudden onset of focal defect of the nervous system without fever or headache. The contrast-enhanced MRI or CT scans may reveal multifocal patchy white matter lesions, with nodular or rim-like enhanced lesions. This is the first case of encephalitis caused by *Mycobacterium rhodesiae* in literature. It is important to note that the definitive diagnosis of NTM encephalitis often relies on a combination of clinical presentation, imaging features, microbiological confirmation, and exclusion of other diagnoses. Due to the variability in presentation and imaging, a high index of doubt is necessary, particularly in patients with risk factors such as immunosuppression. Th clinicians should always consider NTM as a potential cause of CNS infections, particularly when the patient is not responding to standard treatments and in the regions where NTM is more common.

## Data availability statement

The original contributions presented in the study are included in the article/supplementary material, further inquiries can be directed to the corresponding author.

## Ethics statement

This study was approved by the Ethics Committee of Peking University First Hospital. Written informed consent was obtained from the patient and his family for the publication of this case report.

## Author contributions

SC: Formal analysis, Writing – original draft, Writing – review & editing. WS: Investigation, Writing – review & editing. RL: Investigation, Writing – review & editing. LY: Writing – original draft, Writing – review & editing. QP: Formal analysis, Investigation, Writing – review & editing.

## References

[1] Dash A Gupta N Ray Y Kodan P Singh BK Soneja M. Choosing the therapy for neurological infection with rapidly growing mycobacteria. Drug Discov Ther. (2020) 14:211–2. doi: 10.5582/ddt.2020.03026, PMID: 32830168

[2] Velayati AA Farnia P Saif S. Identification of nontuberculous Mycobacterium: conventional versus rapid molecular tests In: Velayati AA Farnia P, editors. Nontuberculous mycobacteria (NTM). New York: Academic Press (2019). 11–59.

[3] Tsukamura M Mizuno S Gane NF Mills A King L. Mycobacterium rhodesiae sp. nov. A new species of rapid-growing scotochromogenic mycobacteria. Jpn J Microbiol. (1971) 15:407–16. doi: 10.1111/j.1348-0421.1971.tb00598.x 5316571

[4] Curry EM Yehia M Roberts S. CAPD peritonitis caused by *Mycobacterium rhodesiae*. Perit Dial Int. (2008) 28:97–9. doi: 10.1177/089686080802800117 18178955

[5] Talati NJ Rouphael N Kuppalli K Franco-Paredes C. Spectrum of CNS disease caused by rapidly growing mycobacteria. Lancet Infect Dis. (2008) 8:390–8. doi: 10.1016/s1473-3099(08)70127-0, PMID: 18501854

[6] Padmanaban V Hussein R Rizk E. Nontuberculous mycobacterial infection in patients with neurosurgical hardware: two cases and a review of the literature. Cureus. (2020) 12:e7398. doi: 10.7759/cureus.7398, PMID: 32337124 PMC7179971

[7] Pacholec M Sami F Newell K El Atrouni W. Fatal disseminated *Mycobacterium haemophilum* infection involving the central nervous system in a renal transplant recipient. J Clin Tuberc Other Mycobact Dis. (2020) 21:100197. doi: 10.1016/j.jctube.2020.100197, PMID: 33294628 PMC7689318

[8] Flor A Capdevila JA Martin N Gavalda J Pahissa A. Nontuberculous mycobacterial meningitis: report of two cases and review. Clin Infect Dis. (1996) 23:1266–73. doi: 10.1093/clinids/23.6.1266, PMID: 8953070

[9] Merkler AE Parlitsis G Patel S Oliveira C Lavi E Schuetz A. Infection of the optic apparatus and hypothalamus by *Mycobacterium haemophilum*. Neurology. (2014) 83:659–60. doi: 10.1212/wnl.0000000000000702, PMID: 25008390

[10] Kon S Franco-Paredes C Hawkins KL. Intramedullary spinal cord lesions in an immunocompromised host due to *Mycobacterium haemophilum*. IDCases. (2020) 19:e00674. doi: 10.1016/j.idcr.2019.e00674, PMID: 32226763 PMC7093745

[11] Leskinen S Flowers X Thoene K Uhlemann A-C Goldman JE Hickman RA. Meningomyeloencephalitis secondary to *Mycobacterium haemophilum* infection in AIDS. Acta Neuropathol Commun. (2020) 8:73. doi: 10.1186/s40478-020-00937-2, PMID: 32430060 PMC7236527

[12] Wallace RJ Silcox VA Tsukamura M Brown BA Kilburn JO Butler WR. Clinical significance, biochemical features, and susceptibility patterns of sporadic isolates of the *Mycobacterium chelonae*-like organism. J Clin Microbiol. (1993) 31:3231–9. doi: 10.1128/jcm.31.12.3231-3239.1993, PMID: 8308116 PMC266383

[13] Gupta N Mittal A VKM N Banerjee S Ray Y Kodan P. Nontuberculous mycobacteria: a report of eighteen cases from a tertiary care center in India. Lung India. (2020) 37:495–500. doi: 10.4103/lungindia.lungindia_365_19, PMID: 33154211 PMC7879861

[14] Herrera D Danyalian A Kaswan D Cohen N Edelstein M Rivero A. *Mycobacterium abscessus* Ventriculoperitoneal shunt infection. Cureus. (2021) 13:e16356. doi: 10.7759/cureus.16356, PMID: 34395133 PMC8359907

[15] Viswanathan R Bhagwati SN Iyer V Newalkar P. Ventriculo-peritoneal shunt infection by *mycobacterium fortuitum* in an adult. Neurol India. (2004) 52:393–4. PMID: 15472442

[16] Giovannenze F Stifano V Scoppettuolo G Damiano F Pallavicini F Delogu G. Incidental intraoperative diagnosis of *Mycobacterium abscessus* meningeal infection: a case report and review of the literature. Infection. (2018) 46:591–7. doi: 10.1007/s15010-018-1141-5, PMID: 29687315

[17] Park M Gupta RK. Central nervous system Mycobacterium infection. Neuroimaging Clin N Am. (2023) 33:105–24. doi: 10.1016/j.nic.2022.07.006 36404038

[18] Kraemer M Berlit P. Primary central nervous system vasculitis – an update on diagnosis, differential diagnosis and treatment. J Neurol Sci. (2021) 424:117422. doi: 10.1016/j.jns.2021.117422, PMID: 33832773

[19] Hedderich DM Lummel N Deschauer M Kümpfel T Schuh E Patzig M. Magnetic resonance imaging characteristics of retinal vasculopathy with cerebral leukoencephalopathy and systemic manifestations. Clin Neuroradiol. (2020) 30:229–36. doi: 10.1007/s00062-018-0755-4, PMID: 30627749

[20] Xie N Sun Q Yang J Zhou Y Xu H Zhou L. High clinical heterogeneity in a Chinese pedigree of retinal vasculopathy with cerebral leukoencephalopathy and systemic manifestations (RVCL-S). Orphanet J Rare Dis. (2021) 16:56. doi: 10.1186/s13023-021-01712-9, PMID: 33516249 PMC7847589

[21] Montero JA Alrabaa SF Wills TS. *Mycobacterium abscessus* ventriculoperitoneal shunt infection and review of the literature. Infection. (2015) 44:251–3. doi: 10.1007/s15010-015-0817-3, PMID: 26148928

[22] Okazaki Y Higashi Y. Unusual cause of progressively impaired cognitive function: *Mycobacterium avium* complex meningoencephalitis. BMJ Case Rep. (2019) 12:e229022. doi: 10.1136/bcr-2018-229022, PMID: 30948407 PMC6453350

[23] Natanti A Palpacelli M Valsecchi M Tagliabracci A Pesaresi M. *Mycobacterium chimaera*: a report of 2 new cases and literature review. Int J Legal Med. (2021) 135:2667–79. doi: 10.1007/s00414-021-02630-y, PMID: 34185152 PMC8523431

[24] Clabots D Serrat A. *Mycobacterium abscessus* peritonitis and ventriculitis associated with ventriculoperitoneal shunt. IDCases. (2022) 27:e01445. doi: 10.1016/j.idcr.2022.e01445, PMID: 35242557 PMC8856985

[25] Wang L Wang F Yang C Luo F. Central nervous system infection caused by *Mycobacterium houstonense*: a case report. Front Neurol. (2022) 13:908086. doi: 10.3389/fneur.2022.908086, PMID: 36119702 PMC9475202

[26] Jung J Shin I Choi Y. A rare case of nontuberculous mycobacterial abscess mimicking brain tumor in an immunocompetent patient. Brain Tumor Res Treat. (2023) 11:219–22. doi: 10.14791/btrt.2023.0019, PMID: 37550823 PMC10409619

[27] Meena DS Kumar D Meena V Bohra GK Tak V Garg MK. Epidemiology, clinical presentation, and predictors of outcome in nontuberculous mycobacterial central nervous system infection: a systematic review. Trop Med Health. (2023) 51:54. doi: 10.1186/s41182-023-00546-4, PMID: 37749661 PMC10518932

[28] Zakrzewski J KimberlyNeisewander BL Esfahani DR Bhimani AD Shah HP Haddadin DW. *Mycobacterium fortuitum* meningitis: approach to Lumboperitoneal shunt infection. South Med J. (2019) 112:217–21. doi: 10.14423/SMJ.0000000000000955 30943540

[29] Haller S Höller C Jacobshagen A Hamouda O Abu Sin M Monnet DL. Contamination during production of heater-cooler units by *Mycobacterium chimaera* potential cause for invasive cardiovascular infections: results of an outbreak investigation in Germany, April 2015 to February 2016. Euro Surveill. (2016) 21:30215. doi: 10.2807/1560-7917.ES.2016.21.17.30215 27168588

[30] Heckman GA Hawkins C Morris A Burrows LL C B. Rapidly progressive dementia due to *Mycobacterium neoaurum* meningoencephalitis. Emerg Infect Dis. (2004) 10:924–7. doi: 10.3201/eid1005.030711 15200833 PMC3323241

[31] Adékambi T Foucault C La Scola B Drancourt M. Report of two fatal cases of *Mycobacterium mucogenicum* central nervous system infection in immunocompetent patients. J Clin Microbiol. (2006) 44:837–40. doi: 10.1128/jcm.44.3.837-840.2006 16517863 PMC1393080

[32] Hwang JH Lee KM Park JE Kim HG Kim EJ Choi WS. Atypical cerebral manifestations of disseminated *Mycobacterium tuberculosis*. Front Neurol. (2017) 8:462. doi: 10.3389/fneur.2017.00462, PMID: 29033887 PMC5627011

[33] Lee MR Cheng A Lee YC Yang CY Lai CC Huang YT. CNS infections caused by *Mycobacterium abscessus* complex: clinical features and antimicrobial susceptibilities of isolates. J Antimicrob Chemother. (2012) 67:222–5. doi: 10.1093/jac/dkr420, PMID: 21980068

[34] Cai R Qi T Lu H. Central nervous system infection with non-tuberculous mycobacteria: a report of that infection in two patients with AIDS. Drug Discov Ther. (2014) 8:276–9. doi: 10.5582/ddt.2014.01047, PMID: 25639308

[35] Wallace RJ Jr Brown-Elliott BA Ward SC Crist CJ Mann LB Wilson RW. Activities of linezolid against rapidly growing mycobacteria. Antimicrob Agents Chemother. (2001) 45:764–7. doi: 10.1128/aac.45.3.764-767.2001, PMID: 11181357 PMC90370

[36] MacGowan AP. Pharmacokinetic and pharmacodynamic profile of linezolid in healthy volunteers and patients with gram-positive infections. J Antimicrob Chemother. (2003) 51:17ii–1725ii. doi: 10.1093/jac/dkg248, PMID: 12730139

